# Local bacteria affect the efficacy of chemotherapeutic drugs

**DOI:** 10.1038/srep14554

**Published:** 2015-09-29

**Authors:** Panos Lehouritis, Joanne Cummins, Michael Stanton, Carola T. Murphy, Florence O. McCarthy, Gregor Reid, Camilla Urbaniak, William L. Byrne, Mark Tangney

**Affiliations:** 1 Cork Cancer Research Centre, University College Cork, Cork, Ireland; 2 Department of Chemistry and Analytical and Biological Chemistry Research Facility, University College Cork, Cork, Ireland; 3 Lawson Health Research Institute, London, Ontario, N6A 4V2, Canada; 4 Department of Microbiology and Immunology, University of Western Ontario, London, Ontario, Canada

## Abstract

In this study, the potential effects of bacteria on the efficacy of frequently used chemotherapies was examined. Bacteria and cancer cell lines were examined *in vitro* and *in vivo* for changes in the efficacy of cancer cell killing mediated by chemotherapeutic agents. Of 30 drugs examined *in vitro*, the efficacy of 10 was found to be significantly inhibited by certain bacteria, while the same bacteria improved the efficacy of six others. HPLC and mass spectrometry analyses of sample drugs (gemcitabine, fludarabine, cladribine, CB1954) demonstrated modification of drug chemical structure. The chemoresistance or increased cytotoxicity observed *in vitro* with sample drugs (gemcitabine and CB1954) was replicated in *in vivo* murine subcutaneous tumour models. These findings suggest that bacterial presence in the body due to systemic or local infection may influence tumour responses or off-target toxicity during chemotherapy.

Tumour responses to chemotherapy vary, and deeper insight into the reasons for therapeutic failure with certain tumours while other apparently similar tumours respond well, stands to guide and improve existing therapeutic regimes, while informing the development of new treatments. Bacteria have been linked with various cancers in a number of ways^1^. For example, local bacterial-induced inflammation has been linked with cancer promotion and progression, via indirect distal effects from the gastro intestinal tract (GIT) microbiome, or directly such as in the case of *Helicobacter pylori*^2^,^3^. Recent research in experimental tumours has revealed that gut bacteria may influence the outcome of chemotherapy indirectly via influencing the immune system^4^,^5^. For decades, naturally occurring bacteria of different types have been isolated from patient tumours of various histological types^1^. In parallel to this, it is well known that deliberate systemic administration of bacteria to animals or patients results in selective replication within solid tumours^6^,^7^,^8^,^9^. Recently, we and others have characterised the bacterial populations naturally present within malignant and non-malignant tissue of the breast^10^. We have reported the presence of a range of bacteria in the breast tissue of cancer patients, while Xuan *et al.* reported similar findings in breast cancer patients, and suggested differences in the types of bacteria present in malignant versus non-malignant adjacent breast tissue^10^,^11^. Others have shown that bacteria such as *E. coli* and *Fusobacterium* are associated with colorectal cancer^12^,^13^,^14^.

Bacteria have the capacity to transform organic chemicals, such as nutrients, pollutants, toxins, drugs and other organic molecules, via endogenous enzymes, and this is exemplified in the field of industrial biotransformation in which various bacteria are used to chemically modify non-biological organic molecules^15^,^16^. Conceivably, there is potential for direct interaction between systemically administered drugs at various body sites in the course of infection or in the case of orally administered drugs and microbiota of the small intestine. So far, the effects of *in situ* bacterial biotransformation of chemotherapeutics, during cancer therapy have not been examined thoroughly. We sought to investigate if bacteria have the potential to influence the efficacy of small drug chemotherapeutics.

## Results

### *In vitro* assay design and validation

**Figure 1:**
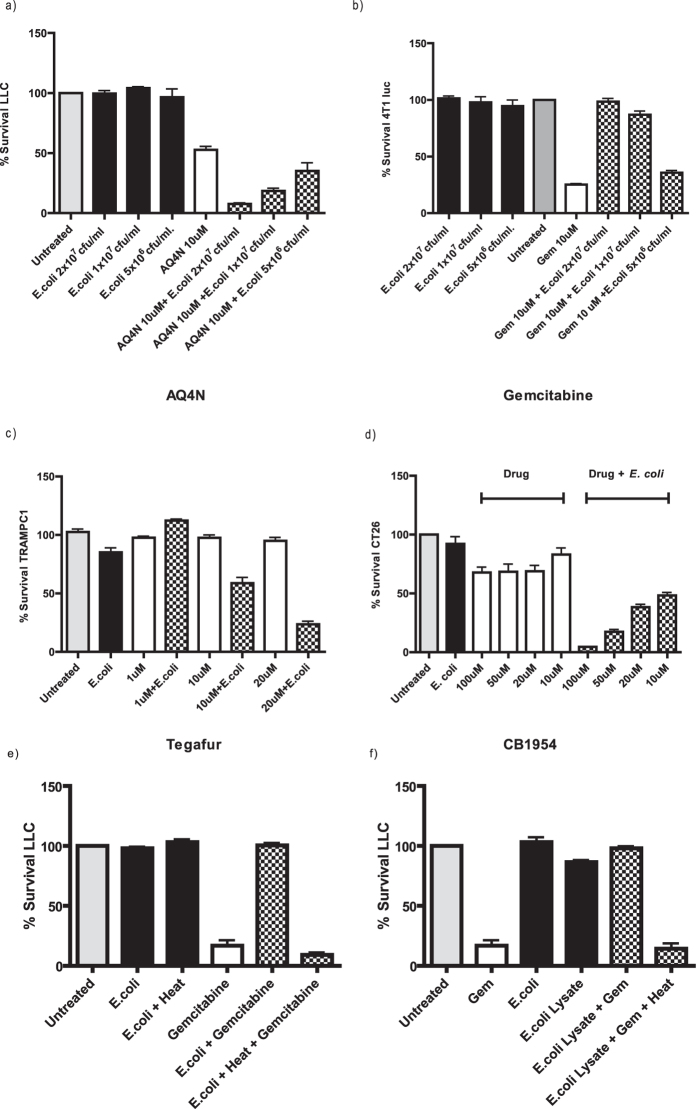
Tumour Cell Survival. Cell survival assay. (**a)** *E.coli* at different cfu/ml were co-incubated with AQ4N (10 μM) after which the supernatant was applied directly to LLC cells (P < 0.01). (**b**) *E. coli* at different cfu/ml were co-incubated with gemcitabine (10 μM) after which the supernatant was directly applied to 4T1 Luc cells (P < 0.01). *E.coli* was co-incubated with Tegafur (**c**) or CB1954 (**d**) at the indicated concentrations after which the supernatant was directly applied to TRAMPC1 cells or CT26 (P < 0.01). Data (**a–f**) represent the average and standard error of four technical replicates. Data shown are representative of 3 independent experiments. (**e**) Tumour cell survival assay stained with MTS. Gemcitabine (10 μM) was incubated with live or heat killed *E. coli* (P < 0.001). (**f**) Tumour cell survival assay. Gemcitabine (10 μM) was incubated with either bacterial lysate (equivalent amounts to cell survival assay live bacteria dosages) alone or bacterial lysate that has been heat inactivated (P < 0.001). Data represent the average and standard error of four technical replicates. Data shown are representative of 2 independent experiments.

Various bacteria and cancer cell lines were examined *in vitro* for changes in the efficacy of cancer cell killing mediated by a range of chemotherapeutic agents. An *in vitro* assay was developed to facilitate drug and bacteria screening as described in experimental procedures and Fig. S1. Based on previously reported microbial analysis of tissue and tumour^10^,^11^ as well as our own observations (Fig. S2) we chose to use *E. coli* as our main drug-testing agent, in addition to *Listeria welshimeri* (see below). Assay validation proceeded with several drugs whose effects were observed to be altered in the presence of *E. coli.* A drug whose efficacy was enhanced (Fig. 1a—AQ4N), and a drug whose efficacy was decreased (Fig. 1b—gemcitabine) by *E. coli* were examined with a range of bacterial concentrations. In both cases, a dose response (P < 0.01) was achieved using three different bacterial concentrations, indicating that changes in drug cytotoxicity were originating from the bacteria. A dose response using different drug concentrations (P < 0.01) was also demonstrated with the drug tegafur and CB1954 (Fig. 1c,d).

In order to investigate if the observed effects were mediated by biochemical modification of the drugs, rather than any physical bacterial drug absorption (e.g. in the case of gemcitabine), the *in vitro* assay was performed with bacteria treated in a number of ways prior to incubation with drug. Gemcitabine was examined with supernatant from *E. coli* which had been lysed by sonication. Similar decreases in cytotoxicity were observed when gemcitabine was incubated with this lysate as with intact bacteria (Fig. 1e,f). Upon heat-inactivation of the lysate (or intact bacteria), no reduction in cytotoxicity was evident (P < 0.001), suggesting the involvement of enzymatic activity.

### Effects of bacteria on chemotherapeutic drug cytotoxicity *in vitro*

Studies proceeded with bacterial strains representing frequently occurring bacterial species in patient tumours; Gram-negative non-pathogenic *E. coli* and Gram-positive *Listeria welshimeri* (also non-pathogenic)^10^,^11^; Fig. S2. Thirty agents from a number of different drug classes were examined, the majority of which represent commonly-employed FDA-licenced drugs. The results are summarised in Table 1. Of 30 drugs examined, the cancer killing efficacies of 10 were found to be decreased, while in general the same bacteria increased the efficacies of 6 others. 14 drugs did not display any difference in efficacy following bacteria co-incubation. Observed effects on drug cytotoxicity were not identical between *E. coli* and *L. welshimeri* (where tested), with *E. coli* increasing the effect of tegafur (unlike *L. welshimeri*), and decreasing the effect of vidarabine, gemcitabine and etoposide phosphate (unlike *L. welshimeri*).

### HPLC and mass spectrometry analyses

**Figure 2:**
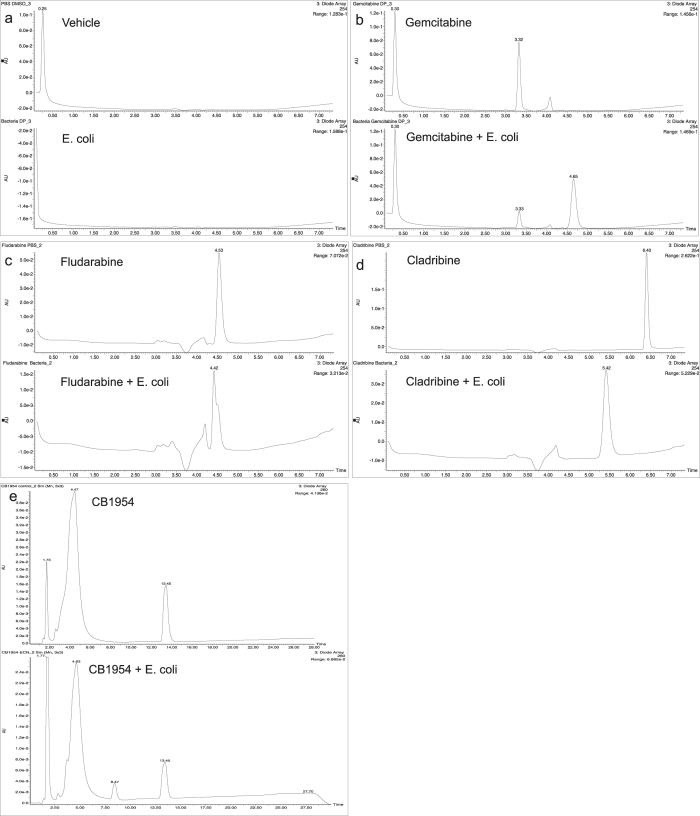
HPLC analysis of drug biotransformations. Chromatograms: (**a**) Top; Vehicle alone (PBS DMSO 0.1%), Bottom; *E. coli* alone (**b**) Top; Gemcitabine, Bottom; Gemcitabine and *E. coli.* (**c**) Top; Fludarabine, Bottom; Fludarabine and *E. coli* (**d**) Top; Cladribine, Bottom; *E. coli* and Fludarabine. (**e**) Top; CB1954, Bottom; E. coli and CB1954 The drugs and their derivatives were detected by UV absorbance at 254 nm.

**Figure 3:**
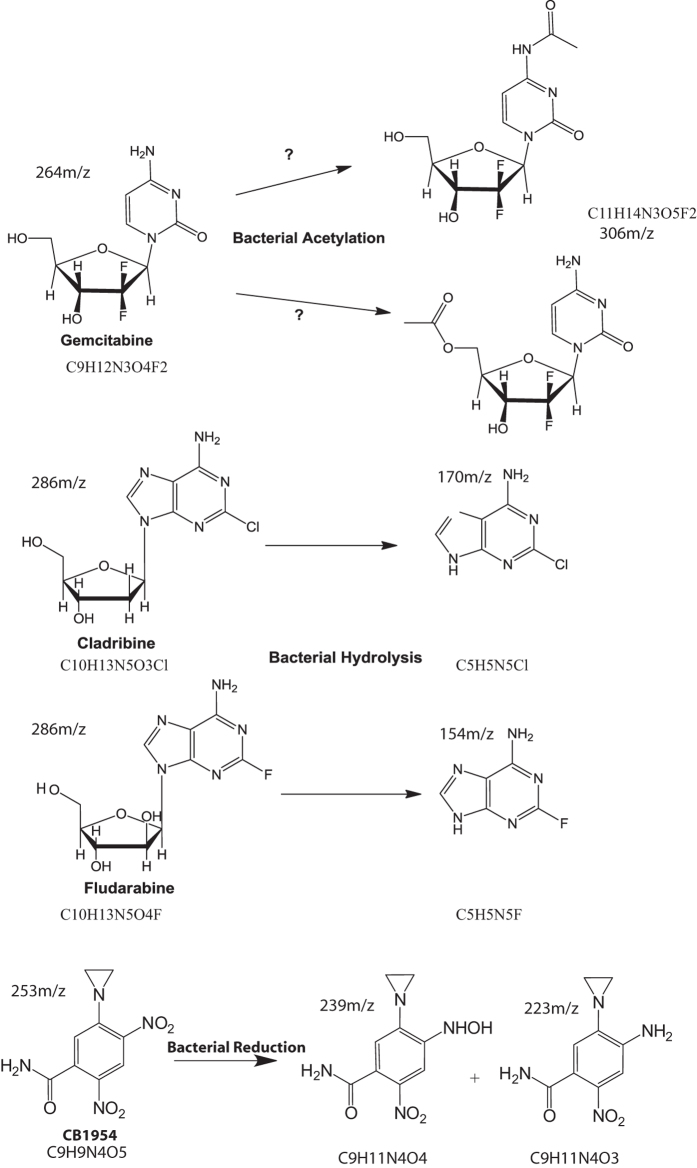
Schematic of drugs and proposed derivatives. Structure prediction of drugs and derivatives based on mass spectrometry analysis. Hypothetical illustrations of structures based on elemental composition analysis and atomic mass fitting of HPLC peaks of drugs or drug derivatives after co-incubation with bacteria. For each molecule, its empirical formula and mass to charge ratio is also shown.

These findings prompted us to analyse a number of sample drugs to elucidate further the basis of drug alteration using HPLC and Mass Spectrometry. All of the drugs tested produced new chromatogram peaks in the presence of bacteria indicating that they were all biotransformed (Fig. 2). Further analysis of each peak by Mass Spectrometry revealed that gemcitabine was biotransformed to a new mass of 306 m/z, (4.65 min) consistent with the formula C_9_H_11_N_3_O_4_F_2_ suggesting acetylation. Fludarabine and cladribine produced new masses of 154 m/z at 4.42 min (C_5_H_5_N_5_F) and 170 m/z at 5.42 min (C_5_H_5_N_5_Cl^35^) respectively, consistent with an enzymatic hydrolysis of the nucleoside’s glycosidic bond yielding the halogenated purine bases. CB1954 (13.45 min) also produced a new peak at 8.47 min in the presence of bacteria which upon further inspection resolved to two mass spectrometry peaks: 239 m/z and 223 m/z equivalent to the published hydroxylamine and amine derivatives that are associated with a CB1954 reduction reaction^17^ (see Fig. 3 for the theoretical molecular structure of the new derivatives).

### Murine models of intratumoural bacterial effects on chemotherapy

**Figure 4:**
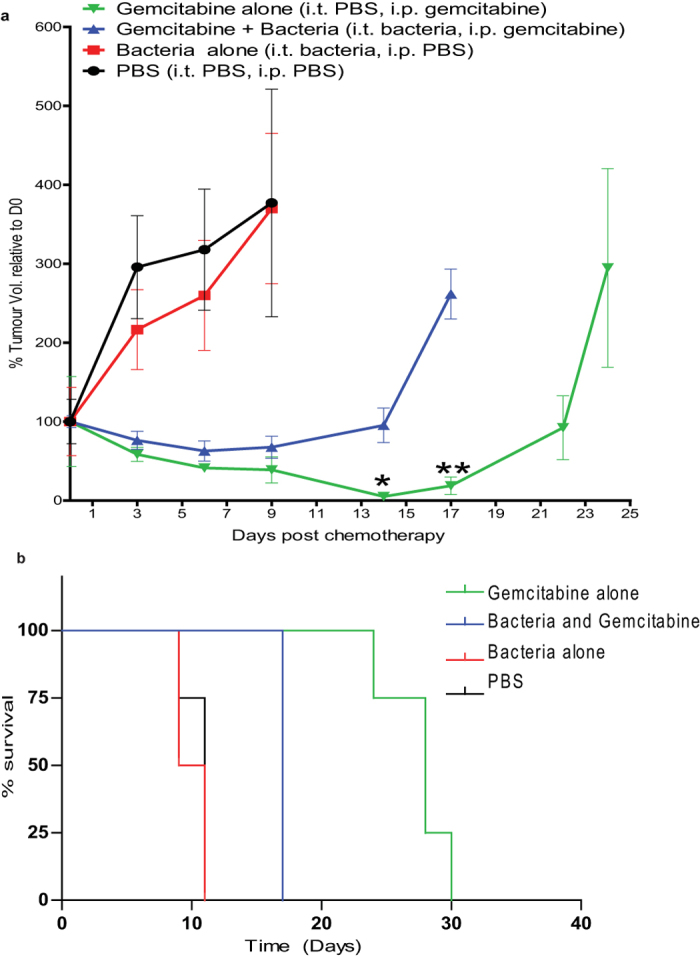
*E. coli* decreases the efficacy of gemcitabine *in vivo.* Subcutaneous flank CT26 tumours growing in Balb/c mice were injected i.t with bacteria or PBS vehicle alone. Gemcitabine (60 mg/kg) was injected i.p. five times at three day intervals. (**a**) Tumour volume (%) relative to the first day of gemcitabine injection (day 0) is shown. *P < 0.03, **P = 0.002 (Bonferroni post hoc test) for gemcitabine alone versus gemcitabine + bacteria. (**b**) Kaplan-Meier plots showing mouse survival over time. The median survival post Day 0 of the gemcitabine + bacteria group was significantly less than that of the gemcitabine alone group (17 days vs. 28 days +/−1.25; P = 0.008). Data are expressed as mean ± SEM of 4 to 8 individual mice per group.

Our lab has previously shown the ability of *E. coli* and other bacterial species to selectively replicate in tumours over time in various murine models^18^,^19^. In this study, we initially validated the growth of *E. coli* in CT26 tumours following intratumoural (i.t.) administration (Fig. S4). The chemoresistance observed *in vitro* (Fig. S3) with a sample drug (gemicitabine) was examined in this model. Mice bearing CT26 tumours were i.t. injected with *E. coli* or PBS, and intraperitoneally (i.p.) injected with gemcitabine or PBS and monitored over time (Fig. 4). As expected, the PBS:PBS and Bacteria:PBS groups showed the greatest tumour volumes over time. Significantly increased tumour volume was observed in the gemcitabine + bacteria group compared with the gemcitabine alone group at various time points (P < 0.03; Fig. 4a). Survival was significantly reduced in the gemcitabine + bacteria group compared with the gemcitabine alone group (17 days vs. 28 days +/−1.25; P = 0.004; Fig. 4b). These data indicate reduced gemcitabine anti-tumour activity in tumours containing bacteria. Bacteria alone did not significantly affect tumour volume relative to PBS administration (P > 0.2), although some reduction is evident in bacteria administered tumours, suggesting that the reduced gemcitabine effect may be partially masked.

**Figure 5:**
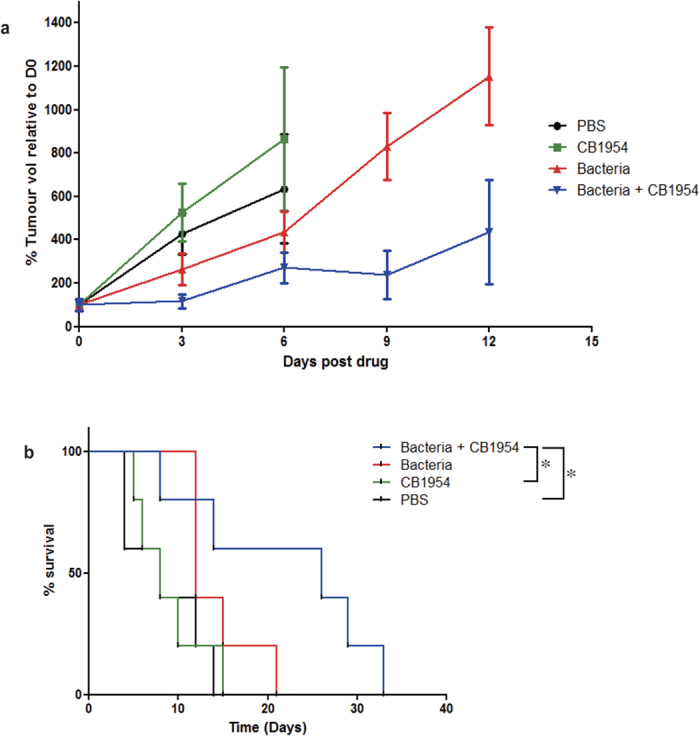
*E. coli* increases the cytotoxicity of CB1954. Subcutaneous flank CT26 tumours growing in Balb/c mice were injected i.t with bacteria or PBS vehicle alone. CB1954 (20 mg/kg) was injected i.p. for the duration of the experiment at 3 day intervals. (**a**) Tumour volume (%) relative to the first day of CB1954 injection (day 0) is shown. (**b**) Kaplan-Meier plots showing mouse survival over time. The median survival post Day 0 of the CB1954 + bacteria group was significantly greater than that of the CB1954 alone group (26 days vs. 8 days. P = 0.0374). Data are expressed as mean ± SEM of 3-5 individual mice per group.

The ability of the same bacterial species to activate the cytotoxicity of another drug, CB1954, was also examined in this model. A significant increase in median survival (26 days vs. 8 days. P = 0.028) was observed in the CB1954 + bacteria group compared with the CB1954 alone group (Fig. 5), indicating drug activation by the intratumoral bacteria.

## Discussion

In this work we examined the ability of wild type bacterial species encountered in the body to influence the efficacy of popular chemotherapeutic drugs that are administered to patients undergoing treatment in the hope of broadening our understanding of bacteria-drug interactions. It is well known that the human body has its own microbiome which differs from one individual to another, with the majority of available information confined to ‘tract’-related body regions^20^,^21^, but also recently placenta, a unique tissue type sharing many features with tumours^22^. Recent data presented by Xuan and co-workers^11^ and our group^10^ describe a multitude of different bacteria taxa living naturally within breast tumours and in the surrounding healthy tissue. The ability of bacteria to opportunistically proliferate or induce infections within tumours of cancer patients has been a sporadic diachronic phenomenon which dates as far back as 200 years^23^. For example, in 1926 Glover stated that certain bacteria were consistently isolated from neoplastic tissue^24^. Between the years 1936–1955 several publications reported that microbes were present in cancer tissue^1^ (it was observations similar to those that ignited interest in using bacteria as anticancer agents leading to the development of Bacillus Calmette-Guérin (BCG) as treatment for bladder cancer^25^). Previous publications detailing sequence analyses of tissue from patients with breast cancer demonstrated the presence of many species, of which *proteobacteria* dominated followed by *fermicutes*^10^,^11^. Furthermore, tissue colonisation as a consequence of cancer chemotherapy is known to occur by antibiotic resistant bacteria^26^.

As we reproduced similar results to Xuan and co-workers^11^ (Fig. S2), we chose *E. coli* (a proteobacterium) and *Listeria welshimeri* (a fermicute) for our studies, because of practical reasons but also because they represent a Gram-negative and a Gram-positive species respectively which have dissimilar outer membranes and metabolism making them attractive for comparative drug studies. Most of the current drug arsenal is composed of small molecular weight organic molecules which are subject to biotransformation (enzymatic modification or degradation) by different tissue enzymes (e.g. cytochrome P450^27^). However, biotransformations can also originate from bacteria which have their own unique ‘enzymolome’.

Initial assay tests were performed with *E. coli* and gemcitabine, AQ4N, tegafur and CB1954 (Fig. 1). Dose responses were achieved with both bacteria and drug as shown in Fig. 1(a–d) and these results validated our *in vitro* assay. While the levels of bacteria used in this assay were arbitrary, such bacterial numbers are not unrealistic in clinical settings^28^. The cumulative data indicate that the effects observed were direct consequences of enzymatic biotransformation. Analysis of gemcitabine suggested that bacterial enzyme(s) was responsible for rescuing cells from its toxic effects as both heat inactivated bacteria or bacteria lysates failed to protect cancer cells in its presence (Fig. 1e,f). Notably, no significant tumour cell death was observed *in vitro* following incubation with supernatant from live or lysed bacteria cells alone suggesting that there is no cytotoxicity arising from any internal toxic metabolite of *E. coli* (Fig. 1e,f).

We decided to screen a panel of drugs which are indicated for various cancers, that would be large enough to cover multiple drug classes to get a better view of the drug modification landscape. However, certain drugs although popular, were omitted from this screen. For example, drugs such as cyclophosphamide, ifosfamide, vinorelbine and vincristine would require a P450 enzyme for modification which is absent in *E. coli* and *Listeria welshimeri*^29^,^30^; the latter also lack the enzyme Carboxypeptidase G2 which is required to degrade pemetrexed^31^; taxanes were unlikely to be modified by our strains as they are artificially produced in bacteria to begin with^32^; finally, drugs such as cisplatin which have an inorganic structure were also omitted. The drug screening was performed in different cell lines where we tested each drug and scored only cases in which we observed a difference between bacteria/drug alone or in combination (Table 1).

The cytotoxicities of approximately 20% of the drugs tested were increased, 30% were decreased and 50% were unaffected. The cytotoxicities of cladribine, vidarabine and gemcitabine and other popular drugs like etoposide phosphate and anti-cancer antibiotics like doxorubicin were decreased by bacteria. The majority of drugs were not affected by the presence of bacteria in our assays, at least at the concentrations examined. The finding that bacteria activated fludarabine and 6-mercaptopurine-2-deoxyadenosine was not surprising. In viral vector-based gene therapy, the enzyme purine nucleoside phosphorylase (PNP) has been used to activate fludarabine when expressed by cancer cells following viral vector delivery^33^. However, the productive interaction of bacteria with a drug in the context of our thesis is a complicated process which may involve both cytoplasmic enzymes and cell membrane transporters before and after a drug’s biotransformation. It may be more relevant to consider a bacterial cell as a reservoir of disparate enzymes or as a biotransformational monad. In agreement with our observations, Chen *et al.*^34^ showed that *Salmonella* can activate the nucleoside 6-methylpurine-2-deoxyadenosine to 6-methylpurine, a cytotoxic agent, intriguingly, despite the fact that fludarabine, vidarabine and cladribine have very similar structures, incubation with bacteria produced opposing effects on cytotoxicity. Similarly, we observed opposite effects with AQ4N (banoxantrone) and its analogue mitoxantrone (Table 1). Consistent with this, Westman *et al.*^35^ also reported that doxorubicin can be biotransformed by *Streptomyces* cell extracts via deglycosylation by NADH dehydrogenase.

In order to gain a molecular insight, we analysed the products of gemcitabine and *E. coli* by HPLC and mass spectrometry and discovered that a new extra peak appeared in the chromatogram which eluted later than gemcitabine (Fig. 2). This new peak had a molecular ion of 306 m/z which indicated that biotransformation had indeed taken place, consistent with a theoretical acetylation at the nitrogen atom of the amine group on the molecule’s base (Fig. 3). However, further molecular analysis would be required to confirm this. The hydrolysis of fludarabine/cladribine to their purine base was not as surprising to witness. Such a reaction is known to be mediated by the isolated enzyme PNP in various gene therapy settings^36^. However, to our knowledge, a productive, direct interaction of live *E. coli/ Listeria welshimeri* with these nucleosides in the context presented in this study (live cell biotransformation and influence of efficacy) has not been reported to date. Especially interesting was the fact that bacteria could hydrolyse cladribine to its chlorinated purine base, but in contrast to fludarabine, they rendered it ineffective (this is also interesting as cladribine is now in clinical trials as an oral drug for multiple sclerosis^37^) The precise molecular mechanism for this difference is currently unknown, but it is relevant to draw attention to the fact that all the therapeutic nucleosides become activated intracellular^38^ so it may be that an extracellular activation of cladribine by *E. coli* along with residual free enzymes from dead bacteria, disables its therapeutic potential. The reduction of CB1954 to its two canonical derivatives shown in Fig. 3 was not a surprise and although this nitroreduction reaction is known to occur, the ability of Gram-positive and negative genetically unmodified bacteria to turn over the prodrug and mediate cell killing has not been shown before. From our screen it becomes apparent that bacteria have the capacity to influence the therapeutic effects of both drugs (e.g. gemcitabine) or prodrugs (e.g. cladribine). The biochemical events which follow after drug activation leading to cell death or drug deactivation are beyond the scope of this project.

We replicated our *in vitro* observations in an experimental mouse model where we demonstrated that bacteria can hamper the effects of a selected drug, focussing on the drug gemcitabine because of its popularity as an anticancer agent and due to our interesting counterintuitive finding that bacteria can neutralise its toxicity. We acknowledge that such a model is quite artificial compared with clinical reality but it provides proof of principle. Nonetheless, it was apparent from the murine data that a growing *in vivo* tumour supports bacterial growth and provides the environment to permit the effects on a sample drug as per the *in vitro* assay (Fig. 4). We further recapitulated the *in vitro* cytotoxicity data from an activating drug (CB1954) in this *in vivo* model (Fig. 5). The *in vitro* (Fig. 1) and *in vivo* data with both drugs examined (in which *E. coli* has opposing effects) are consistent.

Research in this context has primarily focused on the ability of gut microorganisms to affect the metabolism of pharmaceutical agents (reviewed in^39^,^40^). One of the most popular cases studied has been the anti-cancer agent irinotecan and its toxic GIT side effects^41^. The gut bacterium *Eggerthella lenta* deactivates the cardiac drug digoxin influencing its concentration in body fluids^40^. Our findings take the field of microbial-drug interaction a step further, away from the GIT. We have shown that the types of bacteria found in tumours may influence tumour responses to certain chemotherapeutic drugs, either positively or negatively, depending on the characteristics of the infection (different bacteria produced different effects e.g. *E. coli* affected more drugs than *Listeria*). Notionally, drugs can encounter bacteria at different parts of the body (i.e. an infectious focus) in different settings resulting in *in situ* biotransformation. Although bacterial infections do not occur frequently, they are by far not clinically irrelevant: for example, infections of the skin are common^42^ and could induce local skin toxicity if they came in contact with a drug that can be activated to a more toxic form, infections of the stomach by *H. pylori* are common^43^ and can result in the breakdown of L-DOPA (used to treat Parkinson’s disease) affecting its bioavailability^44^; monomicrobial or polymicrobial infections of tissues can arise from blood or solid tumours^44^; bacteraemia due to solid tumours can also present itself in cancer patients^45^ and infections of pleural effusions are not uncommon in thoracic disease of neoplastic origin^46^. Chemotherapy itself has also been associated with increased bacterial infection^47^.

Our findings are also pertinent to alternative anti-cancer strategies that are emerging which investigate combinations of chemotherapy and bacteria to treat solid tumours. Specifically, our work could interest groups working with the clinically approved anti-cancer bacterium BCG which is used to treat bladder cancer^25^. Chemotherapy is used post BCG treatments^48^ or in combination with BCG^49^. In addition to this, early experimental systems that investigate the use of chemotherapy in conjunction with bacteria (Gram-positive or Gram-negative strains)^50^,^51^ should also be aware of potential biochemical drug interactions with their agent’s enzymolome.

In conclusion, our data bring attention to the fact that internal bacteria can interact with a drug therapy and could under certain circumstances influence treatment efficacy and/or side effects.

## Conclusions

Our results show that live wild type bacteria with natural enzyme levels can affect the efficacy of some anticancer agents either positively or negatively *in vitro* and *in vivo*, most likely via enzymatic modifications. Our findings indicate the potential for local or systemic bacterial infections to act as an *in situ* biotransforming reservoirs which may complicate cancer therapy, through reducing anti-tumour efficacy or increasing off-target toxicity. For drugs whose cytotoxicity is increased by bacteria, our data also support the potential to improve therapeutic index through deliberate modification of the bacterial content of cancer patients or tumours.

## Materials and Methods

### Bacteria and cell lines

*E. coli* Nissle 1917 (UCC culture collection) was cultivated aerobically in L-Broth or L-Agar (Sigma) at 37 °C. Bioluminescent *E. coli* was described by us previously^18^ and cultured in the presence of 300 mg/ml erythromycin. *Listeria welshimeri* Serovar 6B SLCC5334 was purchased from ECACC and cultivated at 37 °C in Brain Heart Infusion (BHI) medium. The *E. coli* nitroreductase triple deletion mutant and its parent strain were kindly provided by Dr Antonio Valle^52^. Lewis Lung Carcinoma (LLC), 4T1 (mouse mammary carcinoma) and CT26 (mouse colorectal carcinoma) cells were purchased from ATCC and were propagated according to the supplier’s instructions. The murine recycled prostate cancer cell line TRAMPC1 was kindly provided by Ciavarra RP^53^ of Eastern Virginia Medical School, Norfolk USA, and propagated as described in^54^.

### Drugs

All drugs and enzymes were purchased from Sigma except: Etoposide Phosphate (Santa Cruz), Capecitabine (Santa Cruz), AQ4N (R&D), Nelarabine (A&B), and Vidarabine (Santa Cruz). Drugs were resuspended in H_2_O or DMSO, with appropriate control vehicle utilised accordingly in all experiments.

### Cell cytotoxicity assay

Microtitre plates (96-well) were pre-seeded with 4000 cells/well in appropriate medium for each cell line and allowed to attach overnight. On the day of the assay, bacteria were cultured to log-phase and a subculture ratio, corresponding to an OD_600nm_ of 0.2 determined for each strain, was exposed for 2 h (4 h in the case of IC50s) to drug in falcon tubes containing DMEM in a tissue culture incubator. Falcon content was filter-sterilized to remove presence of bacteria (using 0.2 μm pore filters (Starsted) before adding 200 μl per well. Plates were incubated until cells in control (untreated) had achieved confluent growth. Cytotoxicity was quantified using an MTS staining with the Cell Titre 96 AQueous One solution Cell Proliferation Assay (Promega). The plates were incubated with normal media (80 μl) and MTS solution (20 μl) in a 37 °C incubator for approximately 2 h (until distinct change of colour occurred).

### Bacterial lysis and heat inactivation

Bacteria were heat inactivated at 95 °C for 40 min. Lysis was facilitated by sonication using three 10 sec pulses (at 20 Kz, 50 W). Between each pulse, samples were incubated on ice for 30 seconds. A 20-fold drop in optical density was considered sufficient lysis.

### HPLC and Mass spectrometry analyses

#### Sample preparation

Bacteria were grown to an OD_600_ between 0.8 and 1 in appropriate media. The bacteria were washed once in PBS and then resuspended in the same volume of PBS. The drugs Fludarabine, Cladribine and Gemcitabine were dissolved in DMSO (100 mM) and were added to the bacterial sample to give a final concentration of Fludarabine 10 μM, Cladribine 10 μM and Gemcitabine 1 mM. Bacteria and drug were incubated for 1 h at 37 °C, and centrifuged at 13,500 rpm for 5 min. Supernatant containing drug was transferred to a spin filtration column with a MWCO of 3000 (Vivaspin) and further centrifuged for 10 min at 13500 rpm. Samples were kept on ice before HPLC analysis.

#### *HPLC* and *Mass spectrometry analysis*

The results described were obtained using a Waters Micromass LCT Premier mass spectrometer (Instrument number KD160). Analysis was performed in ESI + mode using a gradient elution method to identify unknowns in the sample. An external reference standard of Leucine enkephalin was infused in order to confirm mass accuracy of the Mass Spectrometre (MS) data acquired. The samples were run in triplicate to ensure consistency and data were analysed by Masslynx 4.1 software. HPLC conditions: A waters Alliance 2695 with a 2996 Photodiode Array detector and Waters Xbridge C18 5 μm 150 × 4.6 mm was used for the chromatographic separation with mobile phase: Acetonitrile (containing 0.1% formic acid) and Water (containing 0.1% formic acid) using the following gradient: 0 min (10:90); 0.5 min (10:90); 5 min (90:10); 10 min (100:0); 11 min (100:0); 11.1 min (10:90); 14 min (10:90). A flow rate of 0.5 ml/min, sample run time of 14 min and injection volume of 1–30 μl was used. For CB1954 the following gradient was used: 0 min (10:90); 0.5 min (10:90); 26 min (30:70); 27 min (10:90); 30 min (10:90). A flow rate of 1 ml/min, sample run time of 30 min and injection volume of 10 μl was used. The MS conditions were as follows: the samples were subjected to ESI + ionisation and acquired from 90 to 1250 m/z at a capillary voltage of 3.00 kV, sample cone of 30 V and a source temperature of 140 °C. An external Enkephalin in Water/Acetonitrile (ESI + m/z = 556.2771) for exact mass correction using Lockspray was used. The UV conditions were set at a sampling rate of 1 spectrum/second, scanning wavelengths from 195–400 nm at a resolution of 1.2 nm.

#### Murine experiments

All animal procedures were performed according to the national ethical guidelines of the Health Products Regulatory Authority (HPRA). Protocols were approved by the University College Cork Animal Experimentation Ethics Committee (AERR #2010/003 and #2012/015).

#### Animals and Tumour Induction

Mice were kept at a constant room temperature (22 °C) with a natural day/night light cycle in a conventional animal colony. Standard laboratory food and water were provided *ad libitum*. Before experiments, the mice were afforded an adaptation period of at least 7 days. Female mice in good condition, without fungal or other infections, weighing 16–22 g and of 6–8 weeks of age, were included in experiments (Harlan, Oxfordshire, UK). At experiment end, animals were euthanised by cervical dislocation. For CT26 tumour induction, 6 × 10^5^ cells suspended in 200 μl of serum-free culture medium were injected subcutaneously (s.c.) into the flank. The viability of cells used for inoculation was greater than 95% as determined by visual count using a haemocytometer and Trypan Blue Dye Exclusion (Gibco), or the Nucleocounter system (ChemoMetec, Bioimages Ltd, Cavan, Ireland). Following tumour establishment, tumours were allowed to grow and develop and were monitored three times weekly. Tumour volume was calculated according to the formula *V* = (*ab*^2^) Π/6, where *a* is the longest diameter of the tumour and *b* is the longest diameter perpendicular to diameter *a*. When tumours reached approximately 100 mm^3^ in volume, mice were randomly divided into experimental groups.

#### Bacterial and drug administration

Overnight cultures of *E. coli* were re-inoculated into fresh LB (1/50 dilution) and incubated shaking at 37 ^o^C until they reached an OD_600_ of 0.7. Cells were then washed twice in PBS. Tumours were administered 10^6^ *E. coli* in an injection volume of 50 μl by intratumoral (i.t.) injection. The viable count of each inoculum was determined by retrospective plating onto LB agar. Two h post bacterial administration, drug (gemcitabine 60 mg/kg or CB1954 20 mg/kg) was administered by intra-peritoneal (i.p.) injection in an injection volume of 50 μl. The drug was subsequently administered on days 3, 6, 9, and 11 and animals not receiving drug were administered an equal volume of PBS vehicle. For imaging experiments, mice were anesthetized via inhalation of isoflurane (Piramal Critical Care, Inc).

### Statistical analysis

For bioinformatics, statistical analysis was performed in R. For biological *in vitro* and *in vivo* assays, two-sided, paired student’s t test with 95% confidence or Mann Whitney U test were employed to investigate statistical differences, using GraphPad Prism or Microsoft Excel 12. Multiple comparison tests were carried out using the Bonferroni post hoc test. Statistical significance of survival between groups in murine experiments was determined using the Log-rank (Mantel-Cox) Test. Survival curves are presented as Kaplan-Meier plots.

## Additional Information

**How to cite this article**: Lehouritis, P. *et al.* Local bacteria affect the efficacy of chemotherapeutic drugs. *Sci. Rep.* **5**, 14554; doi: 10.1038/srep14554 (2015).

## Supplementary Material

Supplementary Information

## Figures and Tables

**Table 1:** Summary of observations from *in vitro* cytotoxicity screen of drugs with bacteria using the bacterial cell kill assay. n/d = not determined, NC = No Change.

Drug Name	Drug Class	Cytotoxicity			
		*E. coli*	*L. welshimeri*		
Tegafur	Anti-metabolite	Up	NC
Fludarabine de phosphate	Anti-metabolite	Up	Up
Capecitabine	Anti-metabolite	NC	NC
5-fluorocytosine	Anti-metabolite	Up	NC
5-fluorouracil	Anti-metabolite	NC	NC
6-Mercaptopurine-2′-deoxyriboside	Anti-metabolite	Up	n/d
Pentostatin	Anti-metabolite	NC	NC
Cytarabine	Anti-metabolite	NC	NC
Clofarabine	Anti-metabolite	NC	NC
Cladribine	Anti-metabolite	Down	Down
Valacyclovir	Anti-metabolite	NC	n/d
Ara G hydrate	Anti-metabolite	NC	NC
Nelarabine	Anti-metabolite	NC	NC
Vidarabine	Anti-metabolite	Down	NC
Gemcitabine	Anti-metabolite	Down	NC
Doxorubicin	Anti-tumour Antibiotics	Down	n/d
Daunorubicin	Anti-tumour Antibiotics	Down	Down
Vinblastine	Anti-tumour Antibiotics	NC	n/d
Actinomycin	Anti-tumour Antibiotics	NC	n/d
Idarubicin	Anti-tumour Antibiotics	Down	n/d
Mitomycin C	Anti-tumour Antibiotics	NC	n/d
Streptonegrin	Anti-tumour Antibiotics	NC	n/d
Etoposide phosphate	Topo Isomerase Inhibitor	Down	NC
Irinotecan	Topo Isomerase Inhibitor	NC	NC
AQ4N	Topo Isomerase Inhibitor	Up	n/d
Mitoxantrone	Topo Isomerase Inhibitor	Down	n/d
Β-Lapachone	Topo Isomerase Inhibitor	Down	n/d
Estramustine	Alkylating Agent	NC	n/d
CB1954	Alkylating Agent	Up	Up
Menadione	Reactive oxygen generator	Down	n/d
