# Supplementary material for: Local bacteria affect the efficacy of chemotherapeutic drugs
Source: Sci Rep. 2015 Sep 29;5:14554. doi: 10.1038/srep14554 (PMC4586607; doi:10.1038/srep14554)
Supplement: Supplementary Information [file srep14554-s1.pdf]

Supplemental Material for the manuscript:

Title: Local bacteria affect the efficacy of chemotherapeutic drugs

Panos Lehouritis[1], Joanne Cummins[1], Michael Stanton[1], Carola T. Murphy[1], Florence O. McCarthy[2], Gregor Reid[3,4], Camilla Urbaniak[3,4], William L. Byrne[1] and Mark Tangney[1*]

[1] Cork Cancer Research Centre, University College Cork, Cork, Ireland

[2] Department of Chemistry and Analytical and Biological Chemistry Research Facility, University College Cork, Cork, Ireland

[3] Lawson Health Research Institute, London, Ontario, N6A 4V2, Canada

[4] Department of Microbiology and Immunology, University of Western Ontario, London, Ontario, Canada

* Correspondence

Mark Tangney PhD

Cork Cancer Research Centre,

BioSciences Institute,

University College Cork,

Ireland

m.tangney@ucc.ie

+353 21 490 1372

Supplementary Figures

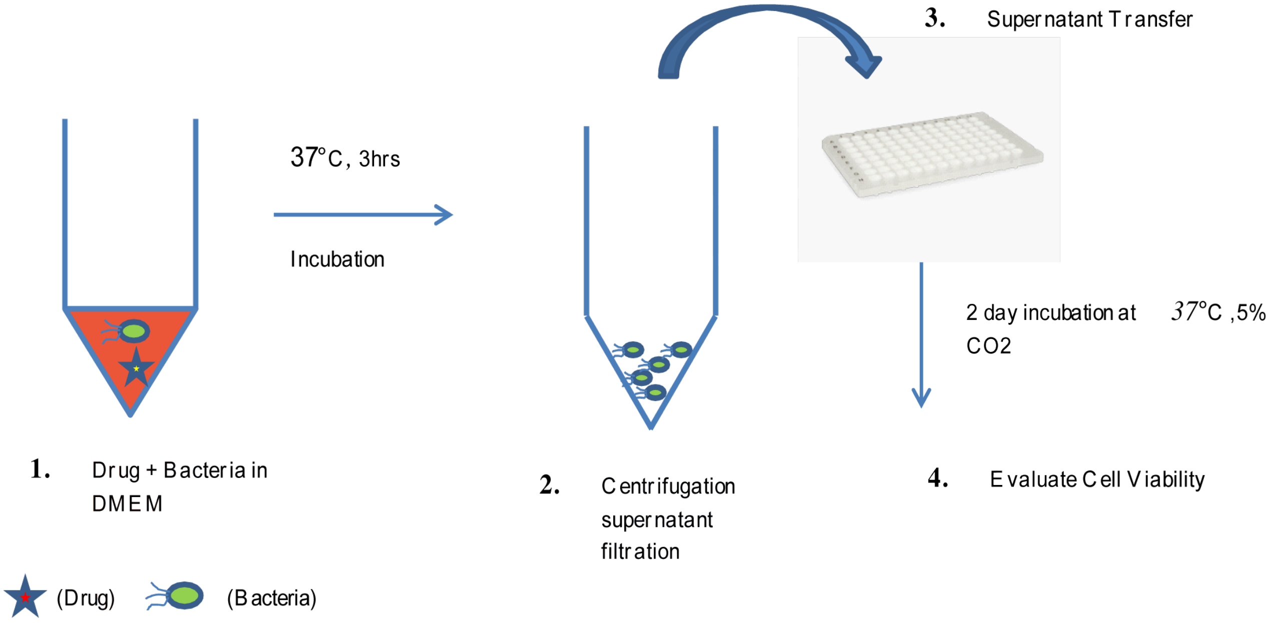

Figure S1: Survival assay illustration **1.** Bacteria and Drug are mixed in DMEM medium, and co-incubated for 3 h at 37°C. **2.** The drug is separated from bacteria using centrifugation and filtration. **3.** The filter sterilised DMEM containing the transformed drug is applied to tumour cells in a 96 well plate. **4.** Tumour cell viability is evaluated via the MTS assay for end point measurements.

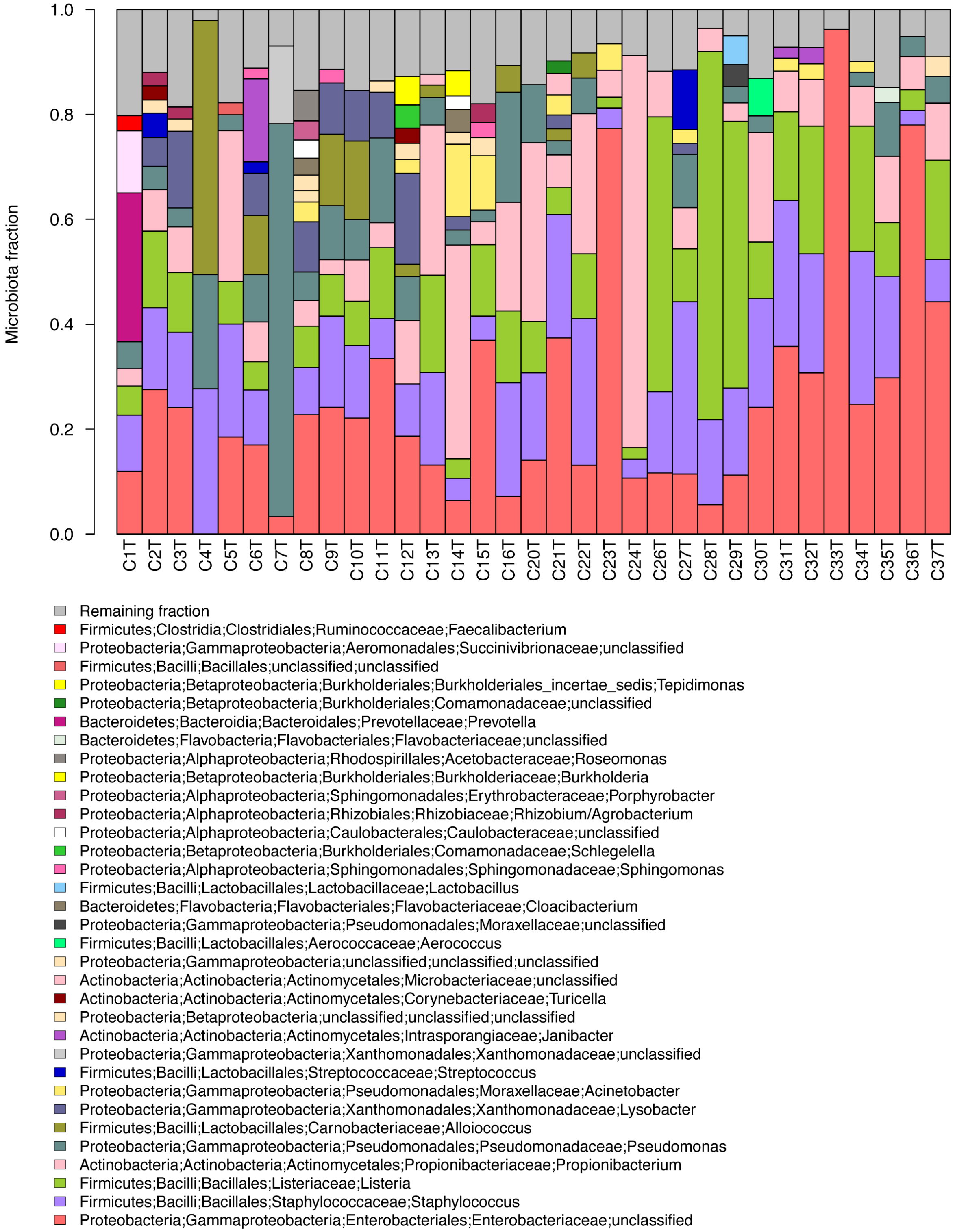

Figure S2: Bacterial content of patient breast tumours Microbiota in tumours identified by 16S rRNA sequencing. The relative abundances of bacterial genera identified in malignant tumour tissue from

33 women were visualized by bar plots. Each bar represents a subject and each coloured box, a bacterial genus. The height of the coloured boxes represents the relative abundance of that organism within the sample. Genera present in less than 2 % abundance in a given sample are displayed in the "Remaining fraction" at the top of the graph (grey boxes). As shown by the barplots, a variety of bacteria were detected within tumour tissue. NB: The legend is read from bottom to top, with the bottom organism on the legend, corresponding to the bottom coloured box on the barplot. If only one species was associated with a particular genus then the species name is also shown in the legend.

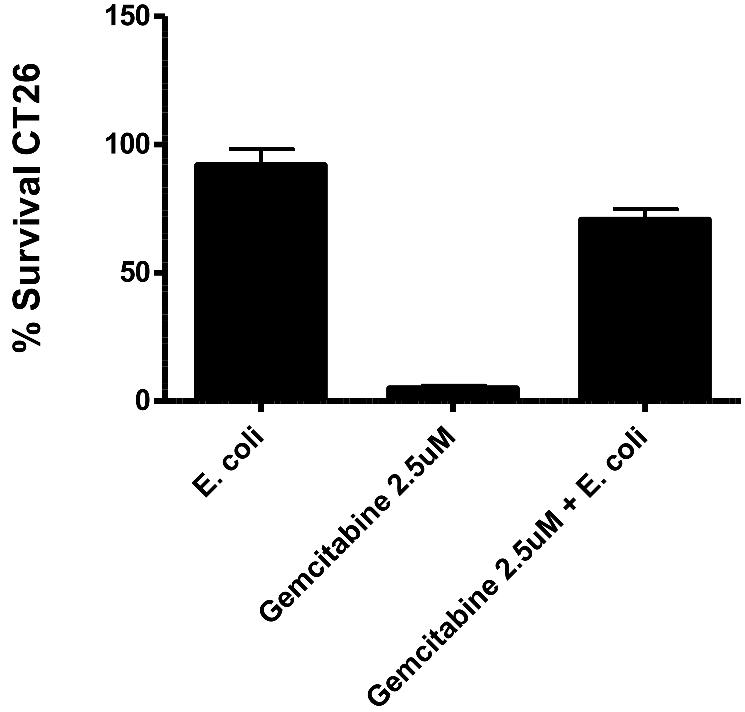

Figure S3: Gemcitabine inactivation by *E. coli* *In vitro* cytotoxic assay using CT26 cells that have been treated with gemcitabine in the presence or absence of *E. coli.* ($P < 0.001$). Data from a representative assay from 3 independent experiments are shown.

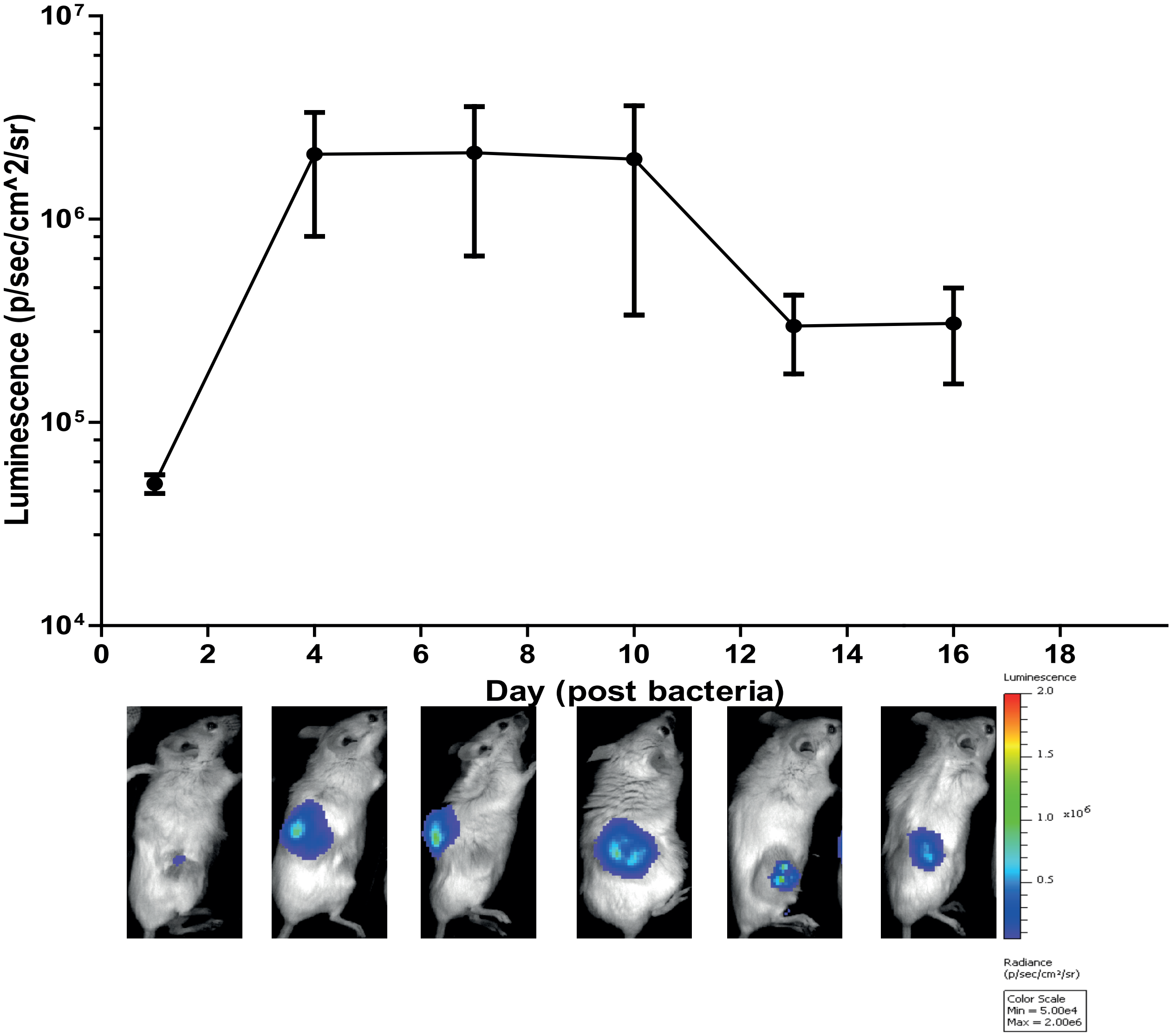

Figure S4: Growth of i.t. administered bacteria in mouse tumours 1×10^6 lux-expressing *E. coli* was i.t. administered to s.c. CT26 flank tumours growing in Balb/c mice. Viable, luminescent bacteria were detected at various time points specifically in the tumour using whole body imaging. A graphical representation of tumour luminescence over time plotted as the mean +/- SEM for four mice, with a representative mouse image under corresponding time points.

Supplementary Materials and Methods

Collection of breast tissue

Ethical approval for this study was obtained from University College Cork clinical research committee and from the patients by informed consent. Breast tumour tissue and corresponding normal adjacent tissue was collected from 33 patients from Cork University Hospital, Ireland with a mean age of 59.8+/-12 years. No patients had received chemotherapy prior to tumour resection. Tumours and normal tissue were surgically removed and harvested under aseptic conditions. Tumour tissue samples were taken from the central region of the tumour and normal adjacent tissue was taken 5 cm from the primary tumour site. Once removed, the specimens were placed in sterile cryotubes and flash frozen in liquid nitrogen within 45 min and then placed at -80 °C until DNA extraction.

Bacterial sequencing

PCR amplification for 16s rRNA gene sequencing: Total DNA was extracted using Gene-Elute Mammalian Genomic DNA miniprep kit (Sigma-Aldrich). A region of 16s DNA was amplified using the barcoded V6-LT primer 5’ CCATCTCATCCC TGCGTGTCTCCGACTCAGNNNNNCWACGCGARGAACCTTACC 3’ and V6-RT 5’ CC TCTCTATGGGCAGTCGGTGATACRACACGAGCTGACGAC 3’ which amplify the V6 hypervariable region of the 16s rRNA gene. The genomic DNA (2.5 μl) was amplified in a 40 μl reaction containing 1.5 mM MgCl, 0.8uM of each primer, 4 μl 10X PCR Buffer, 0.2 mM dNTPs, 0.05U *Taq* Polymerase (Invitrogen) and 0.1 μg/μl of bovine serum albumin. PCR was carried out in an Eppendorf Mastercyler Gradient under the following condition: Initial denaturation at 95 ^{o}C for 2 min followed by 25 cycles of 95 ^{o}C for 1 min, 55°C for 1 min and 72 ^{o}C for 1 min. The presence of a PCR product was verified by electrophoresis on a 2% agarose gel and staining with ethidium bromide. PCR products were stored at -20 ^{o}C until ready for sequencing.

16s V6 rRNA gene sequencing: PCR products were thawed and DNA concentration measured with QuBit 2.0 using the high sensitivity assay. Samples were eluted in 50 µl of buffer and sent to the Robarts Research Institute, University of Western Ontario, London, Canada for Ion Torrent sequencing using standard protocols set up by the facility.

Read processing and taxonomic assignment: Custom Perl and Bash scripts were used to de-multiplex the reads and assign barcoded reads to individual samples. Reads were kept if the sequence included a perfect match to the barcode and the V6 16S rRNA gene primers. Reads were clustered by 97% identity into Operational Taxonomic Units (OTUs) using UClust v. 3.0.617 (*47*). OTUs that represented ≥2% of the reads in at least one sample were kept, while those that did not meet the cut-off were discarded. Taxonomic assignments for each OTU were made by the Ribosomal database project (RDP) SeqMatch tool (*48*). From the top 20 matches to the RDP named isolates database, the full taxonomy was retained for matches with the highest S_ab score. For multiple top matches with equal scores, the highest common taxonomy was retained (e.g. genus level if multiple species matched equally well). Since the maximum number of matches displayed per sequence is 20, the RDP taxonomic assignments were verified by BLAST against the Greengenes named isolates database with an output of 100 hits (*49*). Taxonomy was assigned based on hits with the highest % identities. If multiple hits fulfilled this criterion, classification was re-assigned to a higher common taxonomy. In instances where the highest % identity yielded a single match, if this were less than 90% and the S_ab score from RDP was less than 0.7, taxonomy was assigned at the Family level instead of at the Genus level.
